# Novel Bearing Fault Diagnosis Using Gaussian Mixture Model-Based Fault Band Selection

**DOI:** 10.3390/s21196579

**Published:** 2021-10-01

**Authors:** Andrei S. Maliuk, Alexander E. Prosvirin, Zahoor Ahmad, Cheol Hong Kim, Jong-Myon Kim

**Affiliations:** 1 Department of Electrical, Electronics, and Computer Engineering, University of Ulsan, Ulsan 44610, Korea; andrei.maliuk100@gmail.com (A.S.M.); a.prosvirin@hotmail.com (A.E.P.); cadet.zahoor@gmail.com (Z.A.); 2 School of Computer Science and Engineering, Soongsil University, Seoul 06978, Korea; cheolhong@ssu.ac.kr

**Keywords:** bearing, electric motor, fault diagnosis, feature extraction, feature selection, gaussian window, machine learning, signal processing

## Abstract

This paper proposes a Gaussian mixture model-based (GMM) bearing fault band selection (GMM-WBBS) method for signal processing. The proposed method benefits reliable feature extraction using fault frequency oriented Gaussian mixture model (GMM) window series. Selecting exclusively bearing fault frequency harmonics, it eliminates the interference of bearing normal vibrations in the lower frequencies, bearing natural frequencies, and the higher frequency contents that prove to be useful only for anomaly detection but do not provide any insight into the bearing fault location. The features are extracted from time- and frequency- domain signals that exclusively contain the bearing fault frequency harmonics. Classification is done using the Weighted KNN algorithm. The experiments performed with the data containing the vibrations recorded from artificially damaged bearings show the positive effect of utilizing the proposed GMM-WBBS signal processing to filter out the discriminative data of uncertain origin. All comparison methods retrofitted with the proposed method demonstrated classification performance improvements when provided with vibration data with suppressed bearing natural frequencies and higher frequency contents.

## 1. Introduction

A permanent magnet synchronous motor (PMSM) is a type of synchronous machine with symmetrical three-phase stator windings in which the conventional rotor field windings are replaced with special-shaped rare-earth permanent magnets [1]. PMSMs are widely utilized as generators in renewable energy generation fields such as wind, wave, and tidal power production, as well as in electric motors for cars and heavy-duty transport, such as ship propulsion engines, and for electrification of aeronautical actuation systems [2,3,4]. As a part of various electro-mechanical systems, electric motors are subject to a number of deleterious factors such as heavy-duty cycles, harsh working environments, flawed installations, and factors related to the manufacturing process itself, which may lead to a failure of the electric machine along with the whole system or plant. In [5], the failure source of electrical machines is classified as a combination of different stresses acting on the stator or rotor. In the stationary part, those failure sources are stator thermal stress, stator electrical stress, or stator mechanical and environmental stresses, whereas in the rotary part, those sources are rotor thermal stress, rotor electromagnetic stress, or rotor residual, dynamic, mechanical, and environmental stresses.

**Figure 1:**
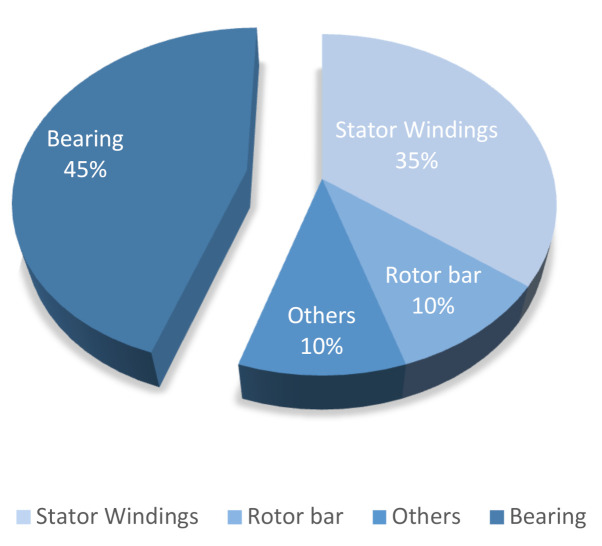
Electric motor fault occurrence.

While there are several sources that can cause the failure of electrical machines, the failures themselves can be classified into two main categories of internal or external depending on the location of the fault source. The causes of internal source failures are manufacturing errors and material deterioration, whereas external faults are caused by interaction with the operational environment, power supply, and load. The internal and external source failures can be roughly split into subcategories such as mechanical, electrical, or environmental, and then into more specific faults types such as rotor strikes, dielectric failure, eccentricity, unbalanced voltage, and poor mounting [6]. The diagram summarizing the components where industrial electric motor faults occur and the occurrence frequency fractions according to reliability studies is presented in Figure 1 [7].

Since PMSMs operate in various complex systems, their failure can lead to degradation of manufacturing quality and extensive damage of the plant, causing significant financial loss and danger to the life and health of the operating personnel. A 45% occurrence of bearing faults in the electric motor compels us to admit the importance of electric motor bearing fault diagnosis study; therefore, the focus of this research work is on electric motor bearing fault diagnosis.

During the history of the development of bearing fault diagnosis techniques, to avoid dangerous accidents related to electric motor faulty operation, breakdown maintenance techniques were replaced with time-based preventive maintenance approaches, which were performed according to working time periods regardless of the true status of the machine. However, nowadays, the application of time-based preventive techniques for modern complex systems is expensive since, to perform the maintenance, the electric motor or even the whole system it is operated in should be stopped; and the expensive system which came up against the maintenance deadline may still have had a significant remaining useful lifetime, but without regard to that, the maintenance would be performed. Therefore, to reduce the cost by lessening the amount of unnecessary scheduled preventive maintenance operations, non-invasive condition-based maintenance approaches are currently considered more efficient; thus, condition monitoring (CM) and precise fault detection and diagnosis systems have become essential for the industry [8]. 

Bearing fault diagnosis using the condition monitoring approach can be successfully solved by either model-based or data-driven techniques. Model-based techniques rely on an accurate model of a system, the development of which is based on a fundamental understanding of the physics behind the process and is expressed in terms of mathematical functional relationships between the inputs and the outputs of the system [9,10,11]. Unlike model-based techniques, the data-driven approaches are based on historical data of the operating process and depend only on the measured process variables. Compared to the model-based methods, data-driven approaches require less design and engineering effort, and with the implementation of modern machine learning techniques, they can easily extract useful information about the system’s current state and greatly contribute to the process of decision making by utilizing data from different sensors attached to the system working in different conditions [12]. 

The bearing arrangement inside the electric motor makes it possible to use different types of source data for fault diagnosis. For instance, Motor Current Signature Analysis (MCSA), traditionally used for detecting and diagnosing electrical types of faults such as stator winding faults, broken rotor bar faults, rotor asymmetry, and abnormal air-gap eccentricity, is now applied for the diagnosis of bearing faults in many works [13,14,15,16,17]. A major advantage of MSCA is that it does not require mounting external sensors for data collection. Another type of data, Acoustic Emission (AE), has been receiving attention due to its ability to detect low-energy signals from low rotation speed bearings with early-stage failure; however, usage of AE data requires working with tremendous amounts of data, which requires a lot of time and computational resources for analysis [18]. Audible sound data have an advantage in collection simplicity due to noncontact sensor installation and are used in research works by Nakamura et al. [19] and Lu et al. [20]. The same noncontact sensor installation advantage along with unique superiorities such as high precision and high sensitivity are the attributes of usage of thermal data from infrared thermography, which is another promising approach for bearing fault diagnosis [21]. Vibration data are traditionally used for bearing fault diagnosis and are currently the industry standard in the area. It is the prevalent method for condition monitoring due to its advantages such as the ability to transfer intrinsic information of mechanical systems and to allow for immediate reaction to changes, which allows it to be used for permanent and intermittent monitoring [22]. Due to this status, vibration signals are considered for bearing fault diagnosis in this work. 

In recent times, data-based bearing fault diagnosis with applications of Machine Learning (ML) has been rejuvenated with a multitude of research. The analysis of those works shows that generally, the bearing condition monitoring approaches consist of two main steps: (1) fault feature extraction and selection and (2) fault classification [23]. Feature extraction and selection is a crucial step since it directly affects the fault classification performance, and thus receives a lot of attention from researchers and industrial professionals, leading to the publication of a significant number of feature extraction methods using various kinds of signal processing techniques. Some of the most commonly used approaches are time-domain statistical analysis methods. Tandon et al. investigated bearing fault detectability using five statistical parameter measurements and performed the analysis of how each feature parameter affected the fault detection performance [24]. The usage of normalized skewness, kurtosis, and third-order statistical moment values from rectified raw data for early bearing fault detection was proposed and investigated by Martin and Honarvar [25,26]. Spectral methods proposed for compensating the shortcomings of Fast Fourier Transform (FFT) such as power spectral density (PSD), second-order displaced power spectral density (SDPSD), signal bispectrum, and Haar wavelets are discussed in [27]. This study showed the ability of SDPSD to reduce noise levels and retain pure harmonic bearing fault signals; however, it could not distinguish narrowband signal resonances from the harmonic signals. The signal bispectrum is more sensitive to phase coupling leaks in the spectrum, which are invisible in PSD. Wavelet analysis based on Haar transform proved to be useful for short transient detection after the periodic signals and noise are eliminated by appropriate filtering. Rubini et al. compared Envelope and Wavelet analysis and also found Wavelet to be more sensitive to the transient phenomena and to the bearing pitting faults after evolution when the contact surface is flattened [28]. The application of the Wavelet Packet Transform (WPT) filter helped to improve the kurtogram ability to denoise and take out the fault features from the signal in comparison with the kurtogram with Short Time Fourier Transform filter. Compared to Morlet wavelet-based kurtogram, WPT achieves simple and fast computation [29]. Spectral kurtosis in combination with the Autoregressive model and minimum entropy deconvolution was proposed by Sawalhi et al. [30]. 

The features of signals extracted using the methods described above are used for the classification of system state. The classification is generally performed by ML techniques. The state-of-the-art supervised ML approaches have gained strong popularity in fault diagnosis at present. In recent times, Deep Learning (DL) techniques have gained in popularity too. As state-of-the-art methods, supervised DL techniques are used for bearing fault diagnosis are Artificial Neural Networks, Convolutional Neural Networks, and Recurrent Neural Networks [31,32,33,34,35]. However, the features extracted by the DL-based methods suffer from a lack of interpretability and the quality of the actual extracted and selected features can be better evaluated by traditional instance-based ML techniques. 

In the research by Rauber et al. [36], the authors refer to an important issue on the formation of the final feature model. They propose a concept of not limiting the feature model to any single one such as, for example, using only wavelet coefficients, but instead suggest using a fusion of features from different feature extraction methods. Their modus operandi is utilizing all the discriminative information no matter where it comes from, as long as it improves the classification performance. The unnecessary or low-quality features can later be eliminated by the feature selection process. 

However, there is another issue that arises before the feature extraction and selection step. The problem of which portion of the time-based signal that should be analyzed for reliable fault diagnosis is pointed out in research work by Kang et al. [37]. In that work, signal processing using Gaussian mixture model (GMM)-based windows is performed to select the fault frequency components from the envelope spectrum and later substitute them into the full envelope spectrum to get the residual signals, which are then used for the Health index calculation and fault classification. Later, Nguyen et al. used the same GMM-windows with health index calculation technique for bearing fault diagnosis using a Deep Neural Network [38]. 

In this paper, the broadened GMM-window-based signal processing approach is proposed to avoid hidden misclassifications that arise from the presence of discriminant information in the data, which is not necessarily related to the actual bearing fault state. Here, for every type of bearing fault, GMM-windows are set for the first ten fault frequency harmonics to reduce the negative effect of possible interference and masking effect inside the selected frequency bands and to suppress the high frequency components. This method allows for extracting reliable features from the selected frequency bands and performs classification with higher accuracy and lighter utilization of computational resources. 

The rest of the paper is organized as follows. The nature of bearing faults and bearing fault frequencies are explained in the remaining Introduction section. Then, the experimental testbed, data collection process, and dataset used in this paper are described in Section 2. The proposed method is presented in Section 3 along with the details of signal processing, feature extraction, feature selection, and classification steps. After that, the results are compared with other methods in Section 4 and the important conclusions are made in the final section of the manuscript. 

### 1.1. Bearing Faults

**Figure 2:**
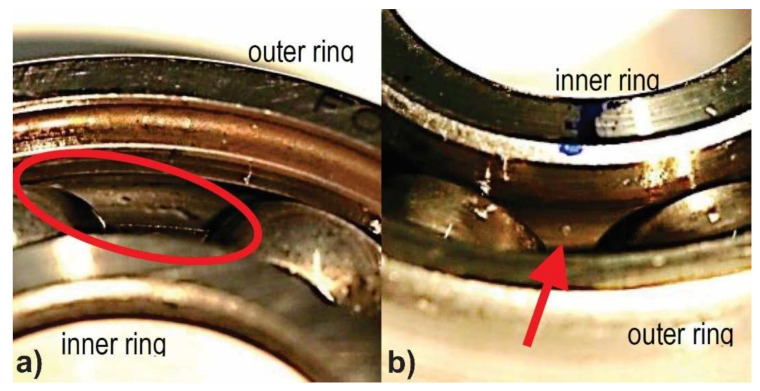
Examples of bearing damage visual representation: (**a**) Indentation at the raceway of the outer ring; (**b**) Small pitting at the raceway of the inner ring.

Electric motors have two sets of bearings at either end of the rotor to support the rotating shaft. These bearings are needed to reduce friction and to enable the rotor to rotate smoothly. Bearings consist of four basic working parts: the outer race, inner race, rolling elements, and separator (also known as the cage in literature). The main factors causing damage to bearings are excessive load, improper lubrication, material fatigue, lubrication contamination, corrosion, and incorrect installation. Bearing damage is visually represented by inner or outer race indentations, spalls, discoloration, or abnormal ball wear path as presented in Figure 2. Generally, bearing faults can be classified as inner race, outer race, or rolling element faults; however, combinations of faults involving several elements of bearing simultaneously are also possible. In this work, only outer race and inner race damage are considered for investigating the capabilities of the proposed methodology. 

During the operation of a bearing with a race fault, rolling elements pass the damaged areas on the race surface. This process generates periodical impulses with a certain rate known as the fundamental defect frequency. These four fault frequencies can be distinguished as the ball spin frequency (BSF), fundamental train frequency (FTF), bearing ball-pass frequency of the outer race (BFPO), and bearing ball-pass frequency of the inner race (BPDI). These characteristic frequencies depend on the geometric parameters of the bearing such as the diameter of the rolling element, the cage diameter, and the pitch diameter, as well as on the number of rolling elements and the angular velocity of the shaft. Equations (1)–(4) for bearing characteristic frequency calculations are given below [16,39]:

(1) $$\mathrm{BSF}=\frac{D_{p}}{2d_{b}}\left(1-{\left(\frac{d_{b}}{D_{p}}\cos\varphi \right)}^{2}\right),$$

(2) $$\mathrm{FTF}=\frac{S_{sh}}{2}\times \left(1-\frac{D_{p}}{d_{b}}\cos\phi \right),$$

(3) $$\mathrm{BPFI}=\frac{N_{b}S_{sh}}{2}\left(1+\frac{d_{b}}{D_{p}}\cos\phi \right),$$

(4) $$\mathrm{BPFO}=\frac{N_{b}S_{sh}}{2}\left(1-\frac{d_{b}}{D_{p}}\cos\phi \right),$$

where *S_sh_* is a shaft speed expressed in revolutions per minute (RPM), *d_b_* is the diameter of the rolling element, and *D_p_* states for the pitch diameter, *N_b_* is the number of rolling elements, and theta is the angle of the load from the radial plane.

**Figure 3:**
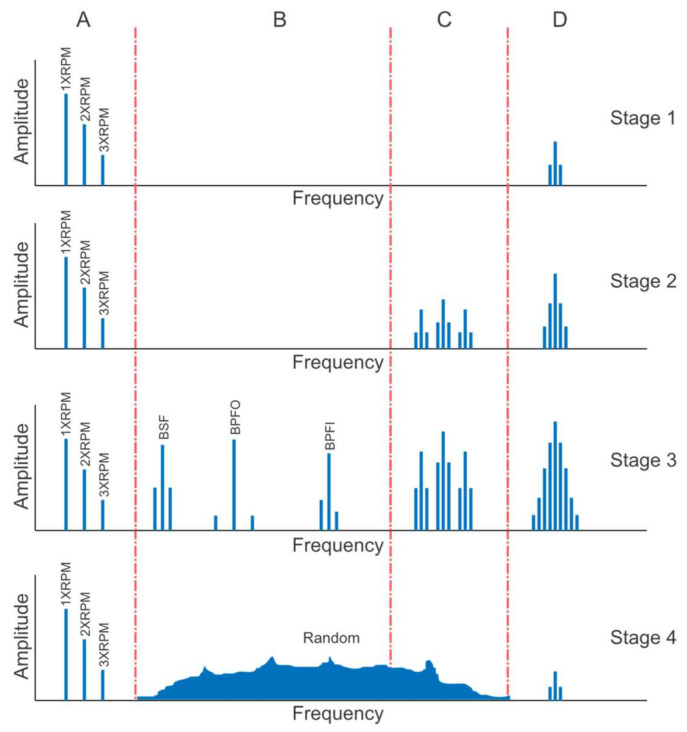
Stages of bearing life.

There are four stages of bearing fault evolution. Every stage is defined by a frequency range and contents. In Figure 3, the bearing vibration frequency spectrum is divided into four ranges. A is the range where the normal components related to speed are present, B is a fundamental bearing fault frequency range, C is a range of bearing natural frequencies, and D is a range for bearing high frequencies. 

In Stage 1, invisible subsurface microcracks are present at ultrasonic frequencies in range D from 20 kHz to 350 kHz. The bearing should not be replaced at this moment. As the wear progresses, the bearing defects start to ring the natural frequency of the bearing components. As a result, in this stage spectral contents start to appear at 500–2000 Hz frequency range C along with higher frequency information in range D. In stage 3, bearing defects become visible. At this stage, bearing fundamental defect frequencies start to appear in range B accompanied by well-formed sidebands and then it is possible to diagnose the damaged bearing component. At stage 4, mainly rotor-related frequencies are present in range A. The bearing damages come to a point where they cause increased rotor vibration. Due to this bearing defect, frequencies decrease in amplitude and more random broadband vibration develops. High frequencies in the range D may grow excessively just prior to bearing collapse. At this stage, the bearing should be immediately replaced [40]. 

## 2. Experimental Setup and Data Collection

**Figure 4:**
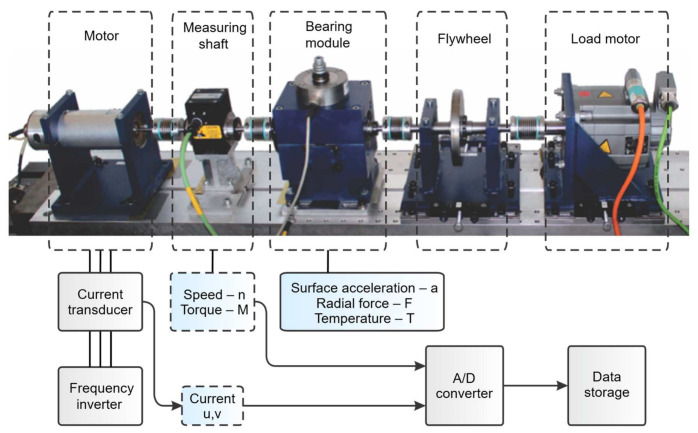
Modular test rig used for data collection.

In this work, the dataset was obtained from the KAt-DataCenter of the Chair of Design and Drive Technology, Paderborn University, Germany [41]. Vibration data in this dataset are collected from the modular test rig shown in Figure 4. The test rig consists of an electric motor, measuring shaft, bearing module, a flywheel, and a load motor. Inside the bearing module, ball bearings with different kinds of damage are mounted for experimental data generation. The electric motor is PMSM type with nominal power of 425 W (Type SD4CDu8S-009, Hanning Elektro-Werke GmbH & Co. KG), which is operated by a standard industrial inverter with a switching frequency of 16 kHz (KEB Combivert 07F5E 1D-2B0A).

**Figure 5:**
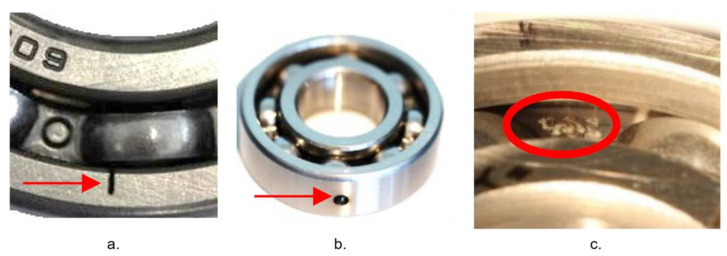
Artificially induced bearing faults: (**a**): EDM trench, (**b**): drilled hole, (**c**): electrically engraved pitting.

For the data acquisition experiment in [41], the authors used healthy and damaged bearings. The tests were performed with 6 healthy bearings with different run-in periods from 1 to over 50 hours and 12 bearings with artificially seeded damage caused by electric discharge machine (EDM), drilling, and manual electric engraving for inner and outer rings. The trenches on the bearing rings inflicted with EDM have a length in the rolling direction of 0.25 mm and 1–2 mm depth. The holes drilled have diameters of 0.9 mm, 2 mm, and 3 mm. The damages inflicted with the manual electric engraver tool are 1–4 mm in length. Visual representations of faults induced by EDM, drilling, and engraving are demonstrated in Figure 5.

Faults of different types with various severity levels were inflicted at the same bearing parts aiming to guarantee the higher robustness of the fault diagnosis methods developed using this dataset. To describe the physiognomy of bearing faults with different parameters, the authors of the dataset introduced a method for general categorization and detailed fault specification partially based on the methodology presented in ISO15243. The method consists of four blocks of information concerning general bearing information, manufacturer-specific information, application-specific information, and information about the damage.

Additionally, the data were collected for 14 bearings with faults obtained by performing accelerated lifetime tests (fatigue damage, damage by plastic deformation, pitting damage, etc.). Totally, 32 different bearings were used to create the dataset. 

Besides inflicting faults of the same type with different levels of severity, another way to ensure the robustness of condition monitoring is to analyze its dependence on test rig operation parameters. For that purpose, the test rig was operated under four different conditions with three varying parameters: rotational speed, load torque, and radial force. Specific parameter values for each of the four operating conditions are presented in Table 1. 

In this work, out of 32 time-series signals available, 18 were used to construct the final dataset. These bearing fault vibration data were collected from artificially damaged bearings. Artificial damages here are represented by single point damages that do not have repetitions and are not combined with any other type of fault. For experimental purposes, the bearing data were arranged in three groups in the following way: six healthy bearings, seven damaged bearings with outer ring faults, and five damaged bearings with inner ring faults labeled as 1, 2, and 3, respectively, as shown in Table 2. 

For vibration data acquisition, a piezoelectric accelerometer (Model No. 336C04, PCB Piezotronics, Inc., New York, NY, USA) and a charge amplifier with a 30 kHz low-pass filter were used. The acceleration was measured at the adapter at the top end of the testbed bearing module, then the signal was digitized and saved with a 64 kHz sampling rate. Each 4 second-length signal provided in the dataset was cut into 1-second pieces, and thus, the complete dataset used for this research is an array with dimensions 5760 × 64,000. 

**Figure 6:**
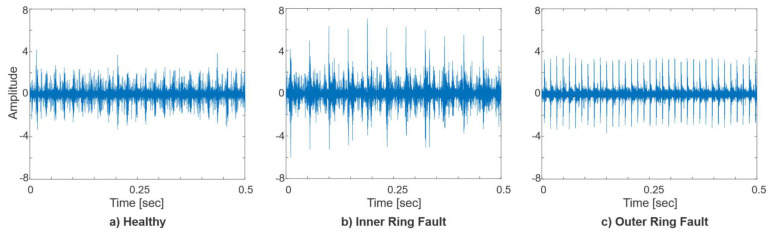
Time-domain raw vibration signal plots of: (**a**) Healthy bearing; (**b**) Bearing with inner ring fault; (**c**) Bearing with outer ring fault.

Time-domain plots of raw vibration signals for a healthy state, inner ring, and outer ring faults are presented in Figure 6. The visual inspection of the plots for preliminary dataset understanding shows that there are noticeable differences between the healthy, inner ring, and outer ring fault signals in time domain representation. 

**Figure 7:**
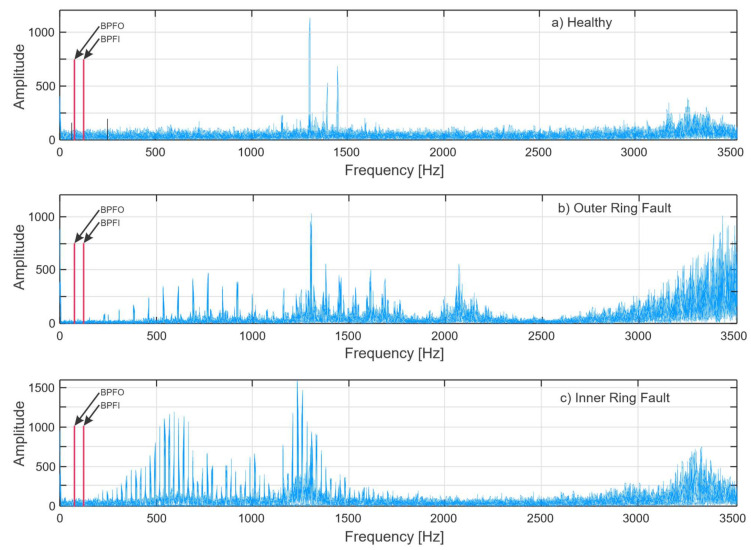
Raw vibration signal spectra of: (**a**) Healthy bearing; (**b**) Bearing with inner ring fault; (**c**) Bearing with outer ring fault.

FFT is a common technique for converting the time domain contents of bearing vibration signal into the frequency domain for spectrum assessment. Figure 7 shows frequency spectra of bearing vibration signals under different health conditions obtained from FFT. For each bearing state, 10 random signals were chosen, and their spectra were plotted for representative comparative analysis of each bearing fault condition. The positions of fault frequency components in Table 3 that are obtained from Equations (3) and (4) are highlighted with red lines on all three bearing fault conditions. However, due to the high-frequency modulation by bearing natural resonance frequencies, BPFI and BPFO are not observable in the frequency spectrum. 

## 3. Proposed Methodology 

**Figure 8:**
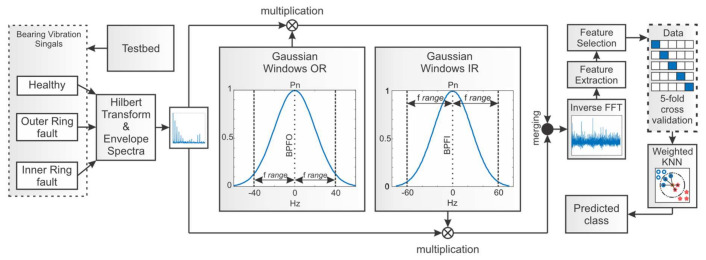
The pipeline of the proposed methodology.

The overview of the proposed methodology is depicted in Figure 8. The whole process is essentially composed of five steps, each of which is described in its separate subsection. First, the Envelope spectra are obtained from raw vibration data by Hilbert Transform and Envelope Analysis. The placement of Gaussian windows along the squared envelope spectra is accomplished based on the computations involving bearing geometric parameters, mechanical parameters of the system, and the rotation speed of the shaft. After the multiplication with GMM-based windows, the obtained signal contains only the bearing fault-related frequency bands. Inverse FFT is performed with the signal to extract 17 features from the time domain. Another three features are extracted from the frequency domain. Then, the feature selection is performed. Selected features are provided to the Weighted KNN algorithm for classification.

### 3.1. Envelope Analysis

**Figure 9:**
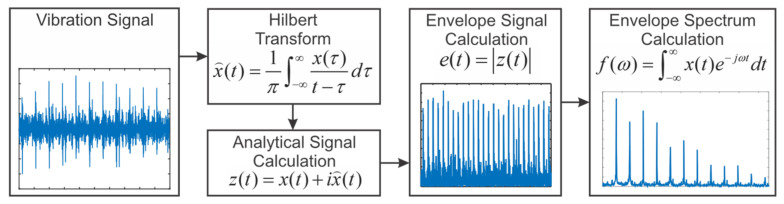
Envelope analysis flowchart.

As shown in Figure 7, the raw vibration signal spectrum does not provide sufficient diagnostic information, because the bearing damage frequencies are amplitude-modulated to the high-frequency region, which results in the bearing fault frequencies observed in the spectrum using the conventional FFT method being visually indistinct. This predicament is resolved utilizing demodulation using envelope analysis which is a prominent signal processing method for bearing diagnostics [42,43]. The flowchart of the Envelope analysis is shown in Figure 9 [18].

Hilbert transform is applied to the bearing vibration signal to calculate the 90-degree phase-shifted signal as follows in the continuous and discrete form:

(5) $$\overset{⌢}{x}(t)=\frac{1}{\pi }\int_{-\infty }^{\infty }\frac{x(\tau )}{t-\tau }d\tau ;\;\;\;\;\;\overset{⌢}{x}(i)=\frac{1}{\pi }\sum_{k=-\infty }^{\infty }\frac{1-{\left(-1\right)}^{k}}{k}x(n-k),\;$$

where *t* is the time, *x*(*τ*) is an input signal sample at *τ,* $\overset{⌢}{x}$(*t*) is a sample of the transformed signal at time *t*, for discrete from *x*(*n*) is the bearing vibration signal. 

Then, the analytical signal is derived as a complex number:

(6) $$z(t)=x(t)+i\overset{⌢}{x}(t)\;,$$

where *z*(*t*) is analytical signal, input signal *x*(*t*) and Hilbert-transformed signal *i* $\overset{⌢}{x}$(*t*) are real and imaginary components, respectively.

The envelope signal $e\left(t\right)$ is then computed as $\left|z\left(t\right)\right|$. Finally, the envelope spectrum, f(ω), was calculated as the square root of the fast Fourier transform of $e\left(t\right)$ as follows:

(7) $$f(\omega )=\int_{-\infty }^{\infty }x(t)e^{-j\omega t}dt;f(k)=\sum_{n=0}^{N-1}x(n)e^{-j\omega n}.$$

It is important to notice that for further analysis, it is often advisable to use the spectra of the squared envelope signal rather than the envelope signal itself. The reason behind it arises from the comparison of spectra of squared signal with the spectra of rectified signal: mathematically, the envelope of a signal is the square root of the squared envelope and the rectified signal is as well the square root of the squared signal. However, application of the square root operator results in extraneous components emerging that are not inherent in the original squared signal. These high harmonic components extend to infinity and appear because of the presence of sharp cusps in the rectified signal. Forasmuch as the whole operation is performed with digital calculations, it is impossible to eliminate the high harmonics using low pass filtration. Consequently, they generate the alias to the measurement range, which causes masking.

**Figure 10:**
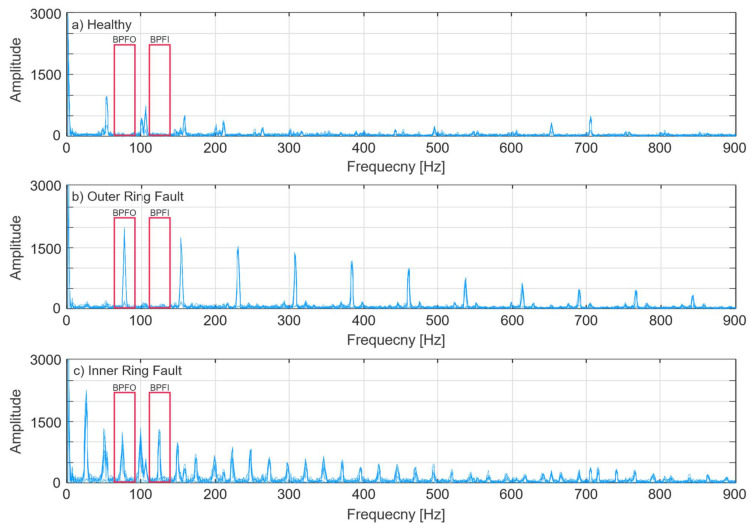
Squared envelope vibration signal spectra of: (**a**) Healthy bearing; (**b**) Bearing with inner ring fault; (**c**) Bearing with outer ring fault.

The squared envelope spectra of a healthy bearing vibration signal, vibration signal of bearing with outer ring faults, and signal with inner ring faults are presented in Figure 10. From this plot, it is evident that in the calculated bearing vibration signal fault characteristic frequencies from Table 3, the BPFO and BPFI components are clearly present; therefore, the bearing vibration signal squared envelope spectrum is further used for the fault feature extraction. 

### 3.2. GMM Window Generation

As discussed in Section 1.1, the squared envelope of the vibration signals contains an abundant amount of information that may be irrelevant to the bearing fault diagnosis task. Until the bearing fault develops to stage 3, there are no fundamental fault frequency components present in the range B, and the components present in the ranges C and D contain information useful for anomaly detection; however, there are no distinctive components that could signify the location of bearing defect. Additionally, bearing normal vibration frequencies are also present in the lower frequency range B and can interfere with bearing fundamental defect frequencies which can complicate bearing fault diagnosis. 

For this case, Gaussian windows are used to select the defect frequency components that are useful for fault diagnosis in each specific application. The parameters of the Gaussian window are determined as follows:

(8) $$\omega_{gmm}(k)=\left\{\begin{array}{l} \sum_{i=1}^{h}\exp\left(-\frac{1}{2}{\left(\beta \frac{\left(k-P_{i}\right)}{\frac{N_{freq}}{2}}\right)}^{2}\right)\\ 0,\;\mathrm{otherwise} \end{array}\right.,P_{i}-f_{range}\leq k\leq P_{i}+f_{range},$$

where *h* is the number of harmonics requiring observation for fault diagnosis task, *P_i_* indicates the *i*th harmonic of the bearing defect frequency, *N_freq_* is the number of frequency bins surrounding each harmonic, and *k* is an index term of each frequency bin, *β* is a coefficient inversely proportional to the standard deviation of Gaussian random variables defined by Equation (10).

The Gaussian window generation procedure in this work consists of the following steps: first, the bearing fault characteristic frequencies for the outer and inner ring faults are calculated for each of the four operating conditions to be considered as the mean for the GMM window. The bearing fault components in the real data are found in the vibration squared envelope spectrum, relying on the results of computations with Equations (3) and (4) and placed in Table 4, Table 5, Table 6 and Table 7. Based on that information, the first point considered for successful Gaussian window generation should be mentioned. In Table 4, Table 5, Table 6 and Table 7, the calculated and observed fault frequencies do not always match exactly and have some random relative error. Thus, aiming to capture the bearing defect harmonics with Gaussian window using Equations (3) and (4), this error is taken into consideration in the window parameter selection process.

**Figure 11:**
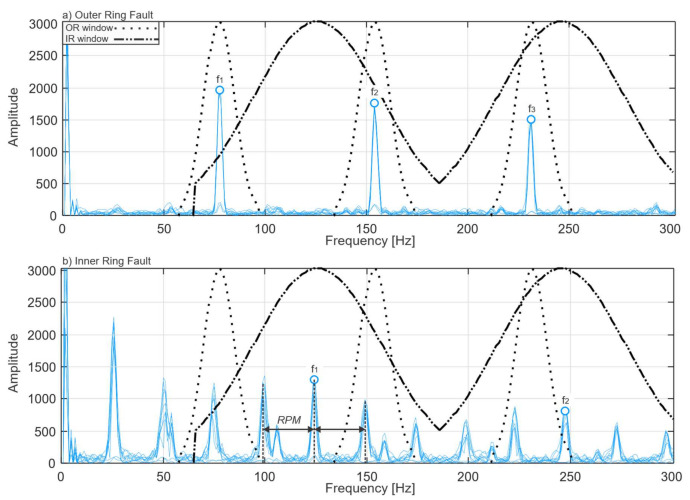
Defect frequency harmonics for: (**a**) Outer ring faults; (**b**) Inner ring faults.

The second point is that the inner and outer ring fault frequency harmonics have different spectral shapes. The radial load influences the force of the impact caused by rolling over a defect; thus, since the outer ring is a stationary component, its defects are subject to the same force at each roll. However, the inner ring rotates at the speed of the shaft, and the defects on the inner ring are subject to varying force; therefore, each harmonic of bearing inner ring fault frequency is amplitude modulated by the rotating speed of the inner ring. This results in the sidebands around BPFI harmonics which are caused by the transitions of the inner ring defect into and out of the load area [44]. Figure 11 demonstrates this difference between the inner ring and outer ring fault envelope power spectra. 

From this figure, it is evident that the envelope power spectrum around the bearing inner ring fault harmonics has a relatively less sharp bell shape due to the presence of the sidebands at both sides of the harmonic at RPM [Hz] distance, whereas the shape of the spectrum around the bearing outer ring fault harmonics is relatively sharp. Therefore, the frequency range for outer ring fault evaluation is chosen to be narrow *f_range_* = 1/4 BPFO, and the frequency range for the inner ring fault evaluation is chosen to be wider *f_range_* = 1/2 BPFI. 

The analysis of frequency spectra of vibration signals with different damage generation methods shows that bearing faults generated by EDM have higher amplitude than drilling faults or faults generated by electric engraver. When comparing the spectra of healthy and faulty signals, it is evident that the spectra of the Inner Ring and the Outer Ring faults generated by drilling and electric engraver contain the characteristic frequency harmonics that can be masked by the harmonics of bearing normal vibration signal; however, even though the drilling and electric engraver generated fault harmonics have lower amplitudes, the profile of the sidebands remains inherent and symptomatic for each bearing fault location. 

Therefore, to prevent possible misclassification of these concurrent harmonics and to achieve better isolation of informative frequency bands containing inherent sideband profiles, the lower and higher-order harmonics containing useful information are segregated using Gaussian windows. Therefore, 10 Gaussian windows are formed to select and explore the first 10 harmonics of the Inner Ring fault signal. The same segregation procedure is performed for the Outer Ring fault vibration signal. As a result of the analysis of several isolated frequency bands, it is possible to distinguish faulty conditions with low signal amplitude even though some of their harmonics can be masked by the healthy bearing vibration frequencies. 

Eventually, the parameters of GMM-based windows are defined as follows:

(9) $$N_{rfreq}=2\cdot f_{range}/f_{resolution},$$

where *f_resolution_* stands for frequency resolution, which in this work is 1 Hz. The coefficient is inversely proportional to the standard deviation of Gaussian random variables and is estimated as follows:

(10) $$\beta =\frac{N_{rfreq}}{N_{wfreq}}\cdot \sqrt{-2\ln\varepsilon },$$

where *N_wfreq_* is the number of frequency bins that are considered as the defect components around the bearing fault characteristic frequency harmonics, and the value *ε* is a constant related to the convergence of Gaussian window which satisfies the condition 0 < *ε* < 1. Adjustment of the *ε* value allows to obtain the necessary window width and flatness according to the needs of the experiment. With the *ε* values close to 0, the window shape will approach a rectangular window. With the *ε* values close to 1, the window shape will approach an all-pass filter. In this study, the best-desired shape of the windows for fault harmonics selection was achieved with *ε* = 0.15.

**Figure 12:**
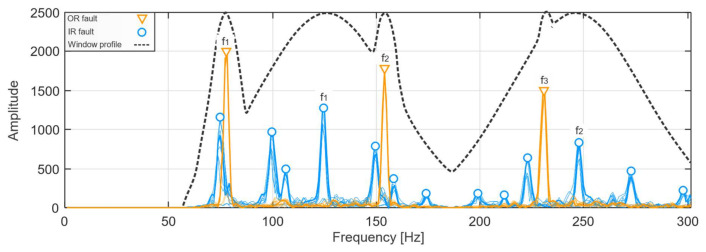
Merged OR and IR signal envelope power spectra after multiplication with OR and IR window sets.

According to the methodology pipeline presented in Figure 8, the results of multiplication with OR and IR windows are merged to yield one signal, containing only the frequency bands that are useful for bearing fault diagnosis. The result of the merging process is presented in Figure 12 for Inner Ring and Outer Ring fault signals and must be considered as the result of the multiplication of signal envelope power spectrum and the set of OR and IR Gaussian windows, which are depicted by a broken line. After this multiplication is performed, the signal is obtained with isolated fault components, which is clearly seen in the signal envelope power spectrum. It is shown in Figure 12, where the blue peaks are the two harmonics of bearing Inner Ring fault signal with their sidebands. The orange trace is bearing the Outer Ring fault signal with three harmonics. 

There is a profuse number of features for dimensionality reduction of time-series data used in various research works in the bearing fault diagnosis field. To make the time-domain feature extraction possible from the signal obtained after the previous steps, the inverse Fourier transform is used to transform the signal from the frequency domain to the time domain. Thus, after the inverse Fourier transform is completed, the time-domain vibration signal envelope containing preselected bearing fault characteristic frequency bands is provided to the feature extraction step. 

### 3.3. Feature Extraction

The bearing fault diagnosis task usually utilizes real-life physical data such as vibration, acoustic emission, or current data. For successful fault diagnosis, a large dataset must be collected describing the state of the system under analysis with different types of faults and operating conditions. There are 96 minutes of signal data in the dataset used in this work and each one-second data sample consists of 64,000 variables (according to the sampling frequency). If raw signals were used for classification, an inordinately large amount of computational resources would be required; however, there would not be any guarantee of good classification results. 

The predicament of the high dimensionality of raw data is resolved utilizing feature extraction. Feature extraction converts data into a set of features called a feature vector, which is a compact informative representation of the data. In this work, 19 statistical features are extracted in total. Specifically, 16 of them are extracted from time-domain sequences and the remaining three are computed from the frequency domain. Those features are widely used in research works on bearing fault diagnosis. The names and equations for them are shown in Table 8 and are listed as peak value, root-mean square, kurtosis, crest factor, clearance factor, impulse factor, shape factor SMR, entropy, skewness, square mean root, 5th normalized moment, 6th normalized moment, mean, shape factor RMS, peak-to-peak value, kurtosis factor, the energy of the signal, frequency center, RMS frequency, and root variance frequency. 

### 3.4. Feature Selection

After the feature extraction process is completed, it is necessary to evaluate the features and eliminate the ones that contain irrelevant or redundant information to prevent the reduction of performance of learning algorithms. This evaluation process is called feature selection. Feature selection aims to form a subset of relevant features with high predictive value, that will allow creating a learning model with the highest possible robustness [45]. 

In this work, feature selection is implemented to speed up the learning and classification processes, avoid overfitting and to increase the accuracy of classifiers, as well as to improve the interpretability of the model by yielding a reasonable number of features that provide the best separation between different classes analyzed in this study. Therefore, feature selection is performed based on the maximum separation distance between different health states of the bearing. This feature selection method is an improved method from [41]. 

Using the notation $j=1,2,\ldots ,N_{f}$ for the given feature set and notation $c=1,2,\ldots ,N_{c}$ for classes of bearing health states, feature selection is performed as follows:

1. The features are normalized between 0 and 1.
2. The mean $\mu_{jc}$ is computed for each feature *f* within class *c* as follows:
  (11) $$\mu_{jc}=\frac{1}{n}\sum_{i=1}^{n}x_{ijc},$$
  where *x* is the feature and *n* is the number of samples.
3. The mean squared Euclidean distance is computed between each feature data point *f* and the mean of the same feature in each class.
  (12) $$d_{f}=\frac{1}{nN_{C}^{2}}\sum_{k=1}^{N_{C}}\sum_{c=1}^{N_{C}}\sum_{i=1}^{N_{C}}{\left(x_{ijk}-\mu_{jc}\right)}^{2}$$
4. The separation distance with the maximum feature separation is normalized to produce a performance evaluation criterion—Normalized Separation Distance (NSD).
  (13) $${\bar{d}}_{j}=\frac{d_{j}}{\max(d)}.$$

**Figure 13:**
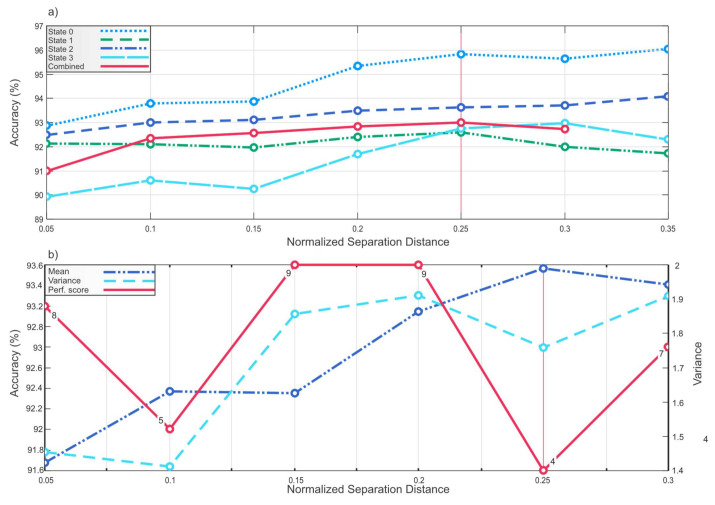
Classification accuracy and NSD value plot; (**a**) Mean value and variance of the classification accuracy for each 0.05 iteration of NSD; (**b**) Accuracy means, variances, and performance scores.

The change of NSD leads to a different set of features being selected. In this work, for every value of NSD starting from zero and increasing by 0.05, a new set of features is obtained. Each set of features that corresponds with each NSD value is provided to Weighted KNN for classification. The classification accuracies are shown in Figure 13. For the feature sets that correspond to NSD higher than 0.4, the classification performance abruptly decreases for some datasets, so the decision was made to ignore the feature sets whose classification accuracy is less than 90% for the convenience of the plot scale and due to the obvious disutility of such feature sets.

The experiments were performed on five datasets: four datasets with different operating conditions (states 0–3), parameters of which are presented in Table 1, and the fifth dataset containing all these operating conditions (combined), which will be further used for training. 

It is evident that feature sets corresponding to the same NSD value do not guarantee improvement of the classification performance for each dataset. Therefore, the NSD value that corresponds to the feature set with the most stable performance on any part of data is chosen based on the following performance stability evaluation method.

For each feature set corresponding to 0.05 iteration of NSD value shown in Figure 13a, mean value and variance of the classification accuracy are calculated using Equations (14) and (15):

(14) $$\mu_{acc}=\frac{1}{n}\sum_{i=1}^{n}x_{iacc},$$

(15) $$\sigma^{2}=\frac{1}{n-1}{\sum_{i=1}^{n}\left(x_{iacc}-\mu_{acc}\right)}^{2},$$

where $x_{iacc}$ is classification accuracy at one threshold for one dataset, $\mu_{acc}$ is the classification accuracy mean for 5 datasets for one NSD value feature set, and $\sigma^{2}$ is the accuracy variance for 5 datasets for one NSD value feature set.

Here, the goal is to choose a NSD value which can provide a feature set that will have the accuracies with the highest mean and lowest variance for all datasets. Hence, means and variances for each examined iteration of NSD value are compared and ranked from one to six in such a way that means are ranked from highest to lowest and variances are ranked from lowest to highest, making the best NSD value have the least sum of rank numbers (performance score) and the worst NSD value the most sum of rank numbers. 

As can be seen from Figure 13b where the accuracy means, variances, and performance scores are plotted, a feature set with the highest mean, lowest variance, and the least performance score corresponds to the NSD value $\bar{d}$*_j_* ≥ 0.25; therefore, this NSD value that corresponds to feature set with the best stable performance for all datasets is chosen for feature selection.

### 3.5. Weighted KNN 

Weighted KNN is chosen as the machine learning classification algorithm due to its instance-based nature. For this reason, the authors consider it an appropriate tool for evaluating the results of frequency band selection and feature selection. 

The selected features are provided as labeled data to the Weighted KNN classification algorithm, which is a nonprobabilistic classification algorithm of the nearest-neighbor type that weighs the evidence of a neighbor close to a new unclassified observation more heavily than the evidence of another neighbor located at a greater distance from the unclassified observation. Specifically, the algorithm uses the weighting function that depends on the distance between the sample and the considered neighbor in such a way that its value decreases with this increase in the distance [46,47]. The weighted KNN algorithm can be described as follows:

1. The training set is given and denoted as:
  (16) $$T={\left\{\left(x_{i}{}^{NN},y_{i}{}^{NN}\right)\right\}}_{i=1}^{N},$$
  where $x_{i}\in {ℜ}^{m}$ is the training vector in the m-dimensional feature space, $y_{i}$ is the corresponding class label, and $x^{\prime }$ is a given query.
2. To start the assignment of a class for the new query $x^{\prime }$, first, the distances of the nearest neighbors of query $x^{\prime }$ are computed for i=1 to *N*:
  (17) $$d(x^{\prime },x_{i})=\sqrt{{\left(x^{\prime }-x_{i}\right)}^{T}(x^{\prime }-x_{i})},$$
3. Then, computed distances of the nearest neighbors are sorted in ascending order forming the set:
  (18) $$T^{\prime }={\left\{\left(x_{i}{}^{NN},y_{i}{}^{NN}\right)\right\}}_{i=1}^{k},$$
4. Out of the set, the search for k-nearest neighbors of the query $x^{\prime }$ is performed for i=1 to *k*:
  (19) $$x_{i}{}^{NN}=x_{sorted\_index(i)},\;y_{i}{}^{NN}=y_{sorted\_index(i)}$$
5. The weights of k nearest neighbors are calculated for i=1 to *k*:
  (20) $$W^{\prime }=\left\{w_{1}^{\prime },\ldots ,w_{k}^{\prime }\right\}$$
  (21) $${w^{\prime }}_{i}=\left\{\begin{array}{cc} \frac{d(x^{\prime },x_{k}^{NN})-d(x^{\prime },x_{i}^{NN})}{d(x^{\prime },x_{k}^{NN})-d(x^{\prime },x_{1}^{NN})}, & \mathrm{if}\;d(x^{\prime },x_{k}^{NN})\neq d(x^{\prime },x_{1}^{NN})\\ 1, & \mathrm{if}\;d(x^{\prime },x_{k}^{NN})=d(x^{\prime },x_{1}^{NN}) \end{array}\right.$$
6. The classification result is computed based on majority weight voting:
  (22) $$y^{\prime }=\arg\underset{y}{\max}\sum_{\left(x_{i}^{NN},y_{i}^{NN}\right)\in T^{\prime }}{w^{\prime }}_{i}\times \delta \left(y=y_{i}^{NN}\right),$$
  where y is an actual class label, $y_{i}^{NN}$ is the class label for the *i*-th nearest neighbor among its *k* nearest neighbors. The Dirac delta function $\delta \left(y=y_{i}^{NN}\right)$ takes a value of one if $y=y_{i}^{NN}$ and zero otherwise.

**Figure 14:**
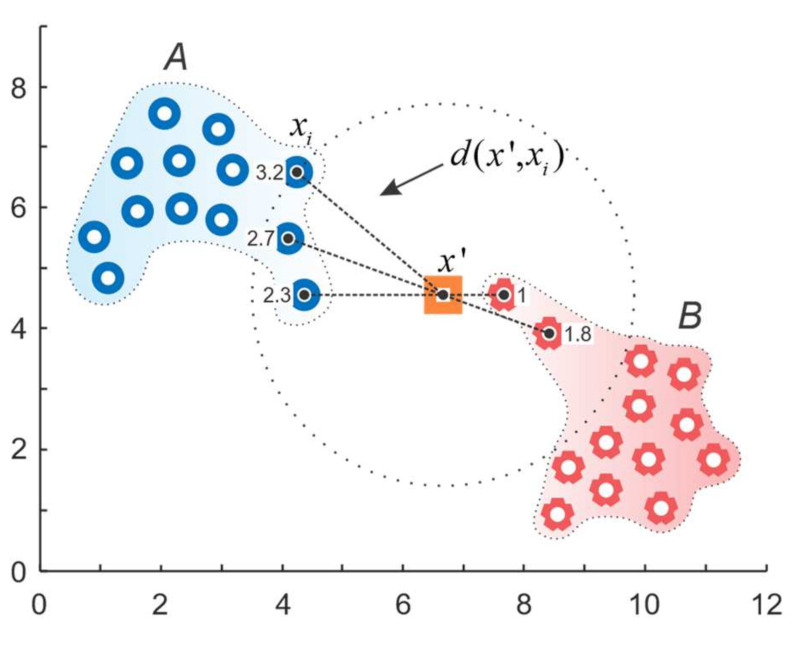
Weighted KNN.

The example in Figure 14 demonstrates the process of Weighted KNN classification. In this figure, the training data set considers only two classes: A and B. A new query $x^{\prime }$ is given to the algorithm and its k (k = 5 for this example) nearest neighbors are identified. The majority voting as in simple KNN algorithm would assign the query $x^{\prime }$ to class A; however, in the case of Weighted KNN, the lesser distances between the new query $x^{\prime }$ and the neighbors from class B add up to a larger weight than the neighbors from class A, and thus, the new query will be assigned to class B.

**Figure 15:**
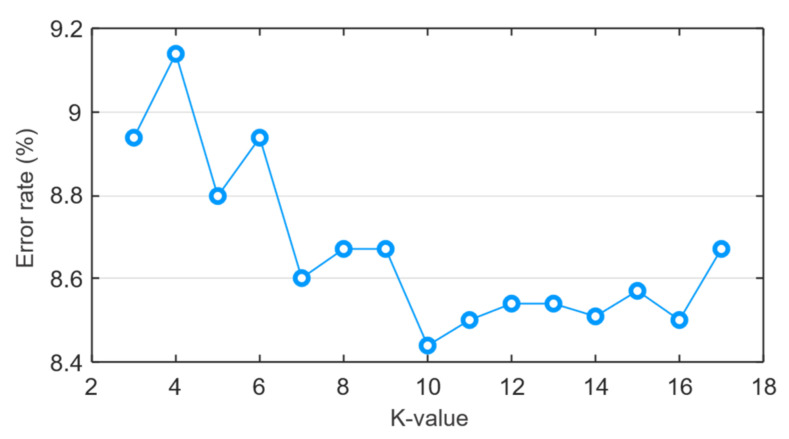
Number of neighbors vs Error rate for the Weighted KNN.

In this work, a weighted KNN model was retrained for the number of neighbors (K-value) from three to 17 with a feature pool without feature selection. Then, the K-value was chosen based on which number of neighbors gave the least classification error rate for Weighted-KNN as is shown in Figure 15. Thus, the number of neighbors was chosen to be 10.

## 4. Fault Identification Performance 

In this section, the proposed bearing fault diagnostic method is evaluated using the vibration data collected from the real testbed that is described in Section 2. First, the algorithm evaluation is performed on 4 different datasets with different states as described in Table 1. Confusion matrices, recall, precision, F1-score, and total fault identification accuracy (FIA) are calculated from the averaged results of 10 experiments performed for each state. The expressions of these metrics are in Equations 23-26:

(23) $$Rec_{\mu }=\frac{\sum_{k=1}^{K}TP_{k}}{\sum_{k=1}^{K}\left(TP_{k}+FN_{k}\right)}\times 100$$

(24) $$Prec_{\mu }=\frac{\sum_{k=1}^{K}TP_{k}}{\sum_{k=1}^{K}\left(TP_{k}+FP_{k}\right)}\times 100$$

(25) F1μ=2×Precμ×Recμ×100/Precμ+Recμ

(26) $$FIA=\frac{\sum_{k}^{K}TP_{k}}{N}\times 100,$$

where $TP_{k}$, $FP_{k}$, and $FN_{k}$ are the true-positive, false-positive, false-negative values computed for the data instances of the class *k*, respectively. *K* denotes the total number of signal classes in the dataset and N states for the total number of data samples in the experimental dataset. The metrics values computed when applying the proposed method to 4 datasets are shown in Table 9. 

**Figure 16:**
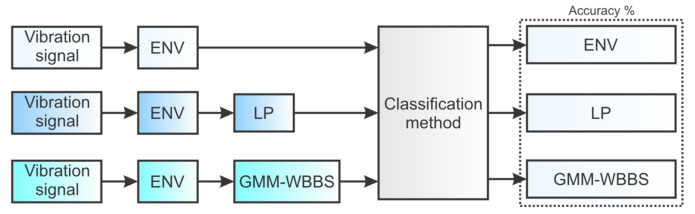
Method comparison pipeline.

Performance comparison with state-of-the-art methods is performed according to the pipeline presented in Figure 16. Each comparison method is substituted into the “Classification method” block. The first method substituted in that block is a method that was used in this work as described in Section 3.3, Section 3.4 and Section 3.5. The second and the third methods are WPT-BE-MSVM and WPT-PCA-MSVM implemented in the same way as described in [48]. Each classification method is trained and tested with the three types of data, which are the same bearing vibration data processed by three types of signal processing techniques. For each method and type of processed data, five experiments were performed.

The reason for such comparison arises from the problem described in Section 1.1 and Section 3.2. For any classifier, the classification accuracy heavily depends on the presence of discriminant information in the data. Signal processing allows the useful content in the data to become more visible and suppresses the signal information that deteriorates the classification performance. Here, the comparison is provided which demonstrates the effectiveness of the proposed method using three types of signal processing approaches:

1. The first approach (ENV) uses the whole vibration envelope squared signal frequency spectrum.
2. The second approach (LP) eliminates bands with possible interconnections and hidden relationships in frequency bands C and D that are useful only for bearing anomaly detection, so the signal contains only the frequency band under 1250 Hz.
3. The third approach is GMM-WBBS. It processes the signal as described in Section 3.2 and outputs a signal that exclusively contains bearing ball-pass frequency harmonics.

Table 10 contains the classification accuracy results for the three state-of-the-art classification methods trained and tested with three types of data obtained after processing the vibration signal by ENV, LP, and GMM-WBBS approaches in columns 1–3. Column 4 contains the difference in classification accuracy between the ENV and LP. The elimination of frequency bands C and D results in a significant performance decrease for each method. The issue hidden here is that provided the whole spectrum, all classification methods erroneously treat bearing natural frequencies and high frequency components as fault signatures, which results in classification accuracy increase; however, as discussed in Section 1.1, the information obtained from frequency bands C and D is useful for bearing anomaly detection on the early stages of the fault. However, those bands provide no insight into the defect location at Stage 2 bearing fault, and thus cannot be used for the diagnosis of the bearing. 

Column 5 contains the difference in classification accuracy between LP and GMM-WBBS. Even though both methods remove frequency bands C and D, classification results using GMM-WBBS are higher for all three classifiers. This difference is a result of the utilization of fault frequency harmonics band selection using GMM-WBBS, which decreases the interference of normal bearing vibration in the lower frequencies and allows for more accurate classification in comparison with simple low pass filtering. 

The difference in classification performance among the proposed method WPT-BE-MSVM and WPT-PCA-MSVM can be explained by the difference in feature selection. Feature selection in the proposed method described in Section 3.4 eliminates the features that do not cross the NSD value, thus leaving only the features with low scatteredness and high interclass separability. PCA, however, does not eliminate features, but instead constructs a lower-dimensional representation utilizing all the given features and does not consider the interclass separability, which in the current situation results in a slightly lower classification compared with the proposed method due to the low quality of the initial feature vector. 

**Figure 17:**
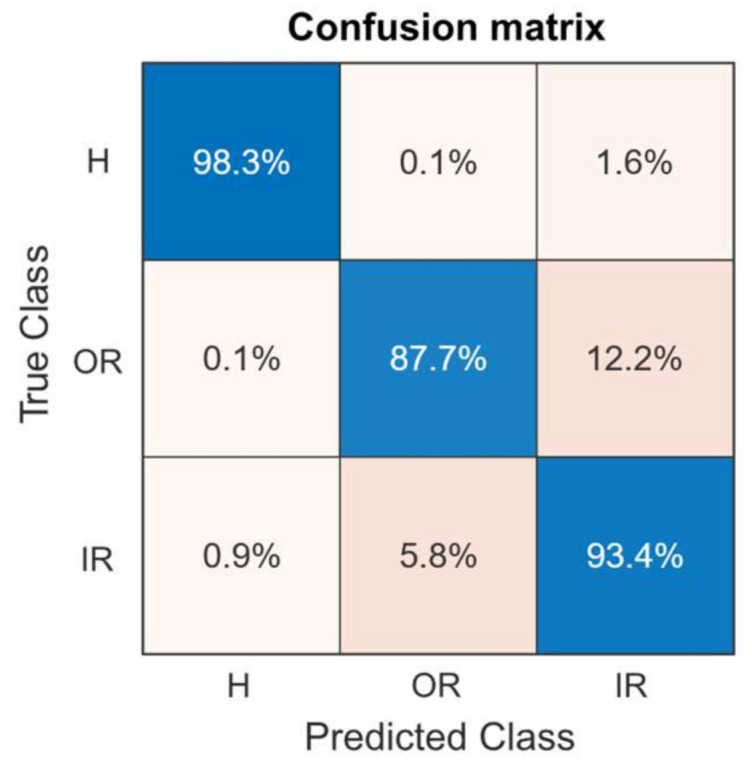
The confusion matrix was obtained using the results achieved by the proposed method.

The confusion matrix for the GMM-WBBS method for all states data with Weighted KNN is provided in Figure 17. The confusion matrix shows the capability of the proposed method to classify healthy bearing, bearing with inner ring fault, and bearing with outer ring fault. The data used in this research contain only single bearing faults, so it is unknown how the proposed method will perform with mixed fault bearing data.

The limitations of the proposed method are within the following factors. Like in most bearing fault diagnosis methods, it is necessary to have the information on RPM at any moment to calculate the fault characteristic frequencies for the GMM-WBBS band selection. The feature selection algorithm uses an NSD value, calculation of which is an iterative process, and which is performed for every bearing operation state with the given data and needs to be improved in the future. Furthermore, in the future, feature extraction quality can be improved by using Deep Learning methods. 

## 5. Conclusions 

In recent years, the burgeoning intelligent bearing fault diagnosis field has attracted a lot of researchers, and the performance of data-driven diagnosis techniques continues to rise. However, at the current moment, a majority of proposed bearing fault diagnosis methods that utilize vibration data do not perform the analysis of the raw data provided to ML algorithms. Thus, the utilized bearing vibration signals may contain discriminative data that may be irrelevant to the actual physical phenomenon of bearing fault. The features extracted from this data can unpredictably both aid or degrade the performance of classifiers. 

Since this problem cannot be resolved by feature selection techniques, a GMM-WBBS method is proposed to address it. To guarantee the immediate relation of the extracted features to actual bearing fault signatures and prevent the use of irrelevant discriminant data, the proposed method exclusively targets bearing fault-related frequency components, that are selected based on construction and parameters of operation. Specifically, the frequency bands in the demodulated bearing vibration signal irrelevant to the bearing fault are suppressed. Then, time domain and frequency domain statistical features are extracted from the demodulated signal containing exclusively bearing fault signature frequencies and the feature selection technique was used to eliminate the low-quality and redundant features. The bearing fault diagnosis was performed using the Weighted KNN classification algorithm. 

The performance of the proposed method is analyzed and compared to state-of-the-art methods. The comparisons demonstrated the effect of utilization of non-relevant discriminative information. WPT-BE-MSVM and WPT-PCA-MSVM methods retrofitted with the proposed GMM-WBBS method show improved classification performance in all cases for fault diagnosis in case of a single fault present. The method was not tested on data with multiple faults occurring at the same time. 

In future work, the feature extraction and selection part of the proposed method can be improved by utilizing Deep Learning techniques. Techniques capable of extracting the RPM information from the vibration data could be beneficial for the simplification of GMM window generation and placement. Besides the improvement of the method, testing the method on data with multiple simultaneous faults will allow it to better evaluate its fault diagnosis capability. 

## Figures and Tables

**Table 1:** Test rig operating conditions.

No.	Rotational Speed (rpm)	Load Torque (Nm)	Radial Force (N)
0	1500	0.7	1000
1	900	0.7	1000
2	1500	0.1	1000
3	1500	0.7	400

**Table 2:** Bearing data arrangement.

Bearing Type	Bearing Code	Class Label
Healthy	K001	1
	K002	
	K003	
	K004	
	K005	
	K006	
Outer Ring Damage	KA01	2
	KA03	
	KA05	
	KA06	
	KA07	
	KA08	
	KA09	
Inner Ring Damage	KI01	3
	KI03	
	KI05	
	KI07	
	KI08	

**Table 3:** Calculated bearing fault characteristic frequencies.

Bearing 6203	State 0	State 1	State 2	State 3
BFPO	76.36	45.81	76.36	76.36
BPFI	123.64	74.19	123.64	123.64

**Table 4:** Calculated bearing fault characteristic frequency harmonics for State 0.

Harmonics	1	2	3	4	5	6	7	8	9	10
BPFO (calculated)	76.36	152.72	229.08	305.44	381.80	458.16	534.52	610.88	687.24	763.60
BPFO (observed)	78	154	231	307	384	460	537	613	690	766
BPFI (calculated)	123.64	247.28	370.92	494.56	618.2	741.84	865.48	989.12	1112.76	1236.4
BPFI (observed)	124	248	371	494	618	741	864	988	1111	1235

**Table 5:** Calculated bearing fault characteristic frequency harmonics for State 1.

Harmonics	1	2	3	4	5	6	7	8	9	10
BPFO (calculated)	45.81	91.62	137.43	183.24	229.05	274.86	320.67	366.48	412.29	458.1
BPFO (observed)	47	93	139	185	231	277	322	369	414	460
BPFI (calculated)	74.19	148.38	222.57	296.76	370.95	445.14	519.33	593.52	667.71	741.9
BPFI (observed)	75	149	223	297	370	444	519	592	666	740

**Table 6:** Calculated bearing fault characteristic frequency harmonics for State 2.

Harmonics	1	2	3	4	5	6	7	8	9	10
BPFO (calculated)	76.36	152.72	229.08	305.44	381.80	458.16	534.52	610.88	687.24	763.60
BPFO (observed)	78	154	231	307	384	460	537	613	690	766
BPFI (calculated)	123.64	247.28	370.92	494.56	618.2	741.84	865.48	989.12	1112.76	1236.4
BPFI (observed)	124	248	371	494	618	741	864	988	1111	1235

**Table 7:** Calculated bearing fault characteristic frequency harmonics for State 3.

Harmonics	1	2	3	4	5	6	7	8	9	10
BPFO (calculated)	76.36	152.72	229.08	305.44	381.80	458.16	534.52	610.88	687.24	763.60
BPFO (observed)	78	154	231	307	384	460	537	613	690	766
BPFI (calculated)	123.64	247.28	370.92	494.56	618.2	741.84	865.48	989.12	1112.76	1236.4
BPFI (observed)	124	248	371	494	618	741	864	988	1111	1235

**Table 8:** Formulas of statistical features extracted from the vibration signal.

Statistical Feature	Formula	Statistical Feature	Formula
Peak value	$X_{p}=\underset{i}{\max}\left|x_{i}\right|$	5th normalized moment	$HOMn5=\frac{\frac{1}{n}\sum_{i=1}^{N}{\left(x_{i}-\mu \right)}^{5}}{{\left(\sqrt{\frac{1}{N-1}\sum_{i=1}^{N}{\left(x_{i}-\mu \right)}^{2}}\right)}^{5}}$
Root-mean square	$X_{RMS}=\sqrt{\frac{1}{N}\sum_{i=1}^{N}x_{i}^{2}}$	6th normalized moment	$HOMn6=\frac{\frac{1}{n}\sum_{i=1}^{N}{\left(x_{i}-\mu \right)}^{6}}{{\left(\sqrt{\frac{1}{N-1}\sum_{i=1}^{N}{\left(x_{i}-\mu \right)}^{2}}\right)}^{6}}$
Kurtosis	$X_{kurtosis}=\frac{1}{N}\left(\frac{\sum_{i=1}^{N}{\left(x_{i}-\mu \right)}^{4}}{\sigma^{4}}\right)$	Skewness	$X_{kurtosis}=\frac{1}{N}\left(\frac{\sum_{i=1}^{N}{\left(x_{i}-\mu \right)}^{3}}{\sigma^{3}}\right)$
Crest factor	$C_{f}=\frac{X_{p}}{X_{RMS}}$	Shape factor RMS	$SF_{RMS}=\frac{X_{RMS}}{\mu }$
Clearance factor	$L=\frac{X_{p}}{{\left(\left(1/N\right)\sum_{i=1}^{N}\sqrt{\left|x_{i}\right|}\right)}^{2}}$	Peak-to-peak value	$x_{ptp}=\max\left|x\right|-\min\left|x\right|$
Impulse factor	$L=\frac{\max\left\{\left|x_{i}\right|\right\}}{\left(\left(1/N\right)\sum_{i=1}^{N}\left|x_{i}\right|\right)}$	Energy of signal	$e=\sum_{i=1}^{N}x_{i}^{2}$
Shape factor SMR	$SF_{SMR}=\frac{X_{SMR}}{\mu }$	Frequency center	$FC=\frac{\int_{0}^{\infty }fs\left(f\right)df}{\int_{0}^{\infty }s\left(f\right)df}$
Entropy	$H(x)=-\sum_{i=1}^{N}P\left(x_{i}\right)\cdot \log_{2}P\left(x_{i}\right)$	RMS frequency	$RMSF=\sqrt{\frac{\int_{0}^{\infty }f_{i}^{2}s\left(f_{i}\right)df}{\int_{0}^{\infty }s\left(f_{i}\right)df}}$
Mean	$\mu =\frac{1}{N}\sum_{i-1}^{N}x_{i}$	Root variance frequency	$RVF=\sqrt{\frac{\int_{0}^{\infty }{\left(f_{i}-FC\right)}^{2}s\left(f_{i}\right)df}{\int_{0}^{\infty }s\left(f_{i}\right)df}}$
Square mean root	$X_{SMR}={\left(\frac{\sum_{i=1}^{N}\sqrt{x_{i}}}{N}\right)}^{2}$		

**Table 9:** Performance metrics values for each operating state.

State No.	Precision	Recall	F1-Score	Total Fault Identification Accuracy
0	95.90	95.90	95.90	95.93
1	92.54	92.54	92.54	92.63
2	93.47	93.47	93.47	93.50
3	92.65	92.65	92.65	92.70

**Table 10:** Classification accuracy comparison.

Method	(1) ENV(acc%)	(2) ENV + LP(acc%)	(3) GMM-WBBS(acc%)	(4) abs(2–1)(acc%)	(5) abs(3–2)(acc%)
Proposed	97.22	91.66	93.1	5.56	1.44
WPT-BE-MSVM	93.60	90.00	91.40	3.60	1.40
WPT-PCA-MSVM	93.08	90.15	91.46	2.94	1.31

## Data Availability

The data of Case Study 1 are publicly available at: https://mb.uni-paderborn.de/kat/forschung/datacenter/bearing-datacenter/data-sets-and-download (accessed on 27 August 2021).
